# Real-Time Location Systems: Revolutionizing Porter Services for Improving Patient Care Efficiency

**DOI:** 10.7759/cureus.69922

**Published:** 2024-09-22

**Authors:** Samriddhi Kataria, Pankaj Sharma, Suryansh Kataria, Meenu Saharan, Rajiv Sikka

**Affiliations:** 1 Independent Researcher, Delhi Public School, Gurgaon, IND; 2 Digital Security, Medanta–The Medicity, Gurgaon, IND; 3 Internal Medicine, Medanta–The Medicity, Gurgaon, IND; 4 Information and Technology, Medanta–The Medicity, Gurgaon, IND

**Keywords:** cost-efficiency, hospital staff, patient satisfaction, porter management, real-time location tracking

## Abstract

Background: Transport porters (TPTs) are essentially involved in supporting hospital operations and maintaining high-quality patient care. The traditional porter management lacks transmission of temporal and spatial information of the porters thus affecting porter operations. In this work, we investigate the barriers in traditional porter management and the impact of a real-time location system (RTLS) in improving time and cost efficiency for porter operations.

Methodology: A six-month study was conducted at Medanta - The Medicity, Gurugram investigating the limitations of traditional management and efficiency of indoor porter tracking system (IPTS) on TPTs operational activities by assessing the waiting time and total turnaround time (TAT). The cost-effectiveness of IPTS was determined based on capital expenditure and return on investment (ROI) post-technology implementation.

Results: This study highlights that improvements are required in porter management as 175 out of 285 (62%) calls were unanswered by TPTs. Post deployment of IPTS, the TAT for A-wing was reduced from 28 minutes to 20 minutes, and for B-wing, it was reduced from 26 minutes to 18 minutes. Staff optimization of three and four porters was done for A-wing and B-wing, respectively. The capital expenditure was recovered by reduced wait time and optimization of staff, thereby anticipating a monthly saving of ₹0.18 million (US$ 2150). The effective outcome of IPTS porter management envisaged its expansion to inpatient medicine delivery with a substantial cost reduction of ₹8.0 million (US$ 96,000) over three years.

Conclusion: The implementation of IPTS improved the operational activities of porters and improved staff utilization. Cost savings from staff optimization lays the groundwork for new technology integrations for long-term operational advancements.

## Introduction

Hospital-based porter services, also known as support service assistants or patient service assistants, play an integral part in patient transport between hospital wards and departments. They are also involved in the transfer of essential supplies, such as medical equipment, linen, blood, and samples [1]. A porter service request by a ward or a department is followed by job dispatch, delivery of service, and confirmation on completion. The smooth functioning of the delivery process is coordinated by the porter team. The porter services, often not recognized as skilled work, are frequently under-managed and under-researched. The lack of attention and management of porter services can lead to inefficient patient care and straining of valuable resources [2].

Earlier studies by Bryan (1998) analyzed integrating porter systems during mergers, and Dershin & Schaik (1993) evaluated the impact of porter services on patient waiting times using queuing theory. A study at the Vancouver General Hospital (VGH) highlighted that delays in delivering patients to CT scan rooms caused by porter unavailability hampered the efficient use of expensive equipment. Considering these critical issues, researchers at VGH performed a two-phase study investigating the porter operations system and proposed improvements for shortcomings [3,4].

Over the years, porter services have been revolutionized with the introduction of hospital porter management systems, especially real-time location systems (RTLS) enabling efficient e-portering, automatic task distribution, and equitable job distribution among the porters. Torkilsheyggi et al. implemented a two-way communication mechanism between nurses and porters to share information about pending patient transport but lacked real-time tracking of porters' location [5]. An explorative study using a mobile application on wearable and handheld devices showed that wearable prototypes effectively supported the maintenance work of orderlies offering domain-specific advantages over handheld devices [6]. Bossen et al. studied the increase in visibility, awareness, and influence of porters after the task management system was upgraded from a manually operated system to a computer-supported system [7]. Although the location information of porters has been used in coordinating the porter services in these works, it is unclear how indoor positioning data were collected, processed, stored, and distributed [2].

The increasing use of electronic health records (EHR) with hospital porter management systems (RTLS) has driven market growth by improving patient care and potentially streamlined patient transport. The global hospital porter management systems market size has anticipated a growth of US$ 103.1 million by 2028 from US$ 54.2 million in 2021, reflecting a compound annual growth rate (CAGR) of approximately 11.3% [8]. RTLS presents a growing opportunity in healthcare through real-time tracking of personnel and resources. The RTLS technology has been deployed in several hospitals in India; however, challenges like cost-effectiveness, data privacy issues, and integration in existing hospital infrastructure have limited its implementation [9,10]. In this study, we investigate the bottlenecks in traditional porter services management, as well as the time- and cost-effectiveness of RTLS in porter management at a multi-specialty tertiary care hospital.

## Materials and methods

Study duration

The study was conducted over a span of six months from June to November 2023.

Site

The study was conducted at Medanta - The Medicity, Gurugram, a Joint Commission International (JCI)-accredited tertiary care hospital in Gurugram, Delhi-National Capital Region. The hospital has a built-up area of over 2 million square feet featuring two identical connected wings in the east and west, called the A and B wings. A hospital patient's journey involves visits to various departments in the hospital like the laboratory, radiology, cardiac science, pulmonary, urology, and many more. These constant patient movements between departments demand wheelchair transport across multiple floors. Elevators are utilized for transitions between departments on different floors. Patients enter and exit through the main lobby foyer on the ground floor. Porter services aid in the smooth functioning of the hospital and in a specific patient's journey from start to finish. Four base stations or "pools" located at the upper ground, fourth, seventh, and 14th floors serve as the dispatch centers for these porters.

Traditional porter management

Nurses raise a request for a transport porter (TPT) through a unique communication phone number, which gets directed to the nearest base station. The base station dispatches a TPT to the designated ward, where the ward nurse provides the TPT with an assigned location. The TPT then carries out the assigned patient transportation task.

The transportation task for a TPT may be allotted at the pool or self-assigned on request. Total turnaround time (TTT) refers to the time between a porter being assigned a task to the completion of the task, for example, the patient arriving at the destination location. Medanta Hospital has a porter fleet of 91 porters. The distribution of these porters' deployment is given in Table 1. The operational timings for TPTs are divided into two shifts of 12 hours and eight hours.

**Table 1:** Distribution of transport porters deployed at the hospital (n = 91). IPD: inpatient department; OPD: outpatient department; TPT: transport porter.

Approved porter deployment	Manpower
Manager	1
IPD supervisor	1
Main porch supervisor	1
Team lead (TL)	6
TPT IPD pool No. 1	11
TPT IPD pool No. 2	12
TPT IPD pool No. 3	13
TPT IPD pool No. 4	14
TPT main porch pool	14
TPT night pool	11
Pharmacy pool	2
Physiotherapy pool	2
3^rd^ Echo	1
9^th^ Echo	1
8^th^ OPD	1

The inpatient department has separated porter pools to maximize the efficiency of patient transfers. These porters are further allotted to different specialties, as shown in Table 2.

**Table 2:** Further allotment of IPD porters into specialties. IPD: in-patient department; OPD: outpatient department; EHC: executive healthcare; TPT: transport porters; ECG: echocardiogram; OBG: obstetrics & gynecology; PAC: pre-anesthesia checkup; TMT: treadmill test; DSE: dobutamine stress echocardiography.

Floor	Specialty	Approved manpower
2nd floor	2nd OPD	1
2nd floor	EHC, Wellness Center, PAC clinic (TMT, ultrasound, stress echo, sample collection, ECG), dental	7
3rd floor	Cardiology	4
3rd floor	DSE, echo, ECG, nursing assessment	1
4th floor	Respiratory & chest surgery	3
5th OPD	Endocrinology, vascular, derma, rheumatology	3
6th OPD	Neurosciences	3
7th OPD	Muscular-skeleton	4
8th OPD	Gynecologic oncology, ophthalmology, ENT, plastic	4
9th OPD	Internal medicine, pediatrics, OBG	2
10th OPD	Medical oncology, radiation oncology, breast services & chemo daycare	7
11th OPD	Gastrointestinal	2
12th OPD	Nephrology, urology, urodynamics	4
14th OPD	Robotic urology, liver transplant	2

The traditional management system does come with its share of cons. The facility being decentralized in nature restricts the ability of TPTs to meet transport requests promptly. Locating the porters was time-consuming due to a lack of real-time trackers. Computer-based porter management like real-time location systems (RLTS) and indoor porter tracking systems (IPTS) have been proposed to address the need for coordinated porter services in hospitals. The implementation of RTLS needs huge capital expenditure for live tracking and automatic flow management in the form of deploying beacons or internet of things (IoT) gateways over entire pathways. These gateways especially focus on the hot zones where the traffic is greatest like the lab, radiology, or cardiac departments, etc. Therefore, before deploying a full-fledged IPTS, a field trial was conducted for three weeks to collect daily operations of porter services in a hospital and identify the pain points in transporting patients.

Architecture of the in-building porter tracking system

**Figure 1:**
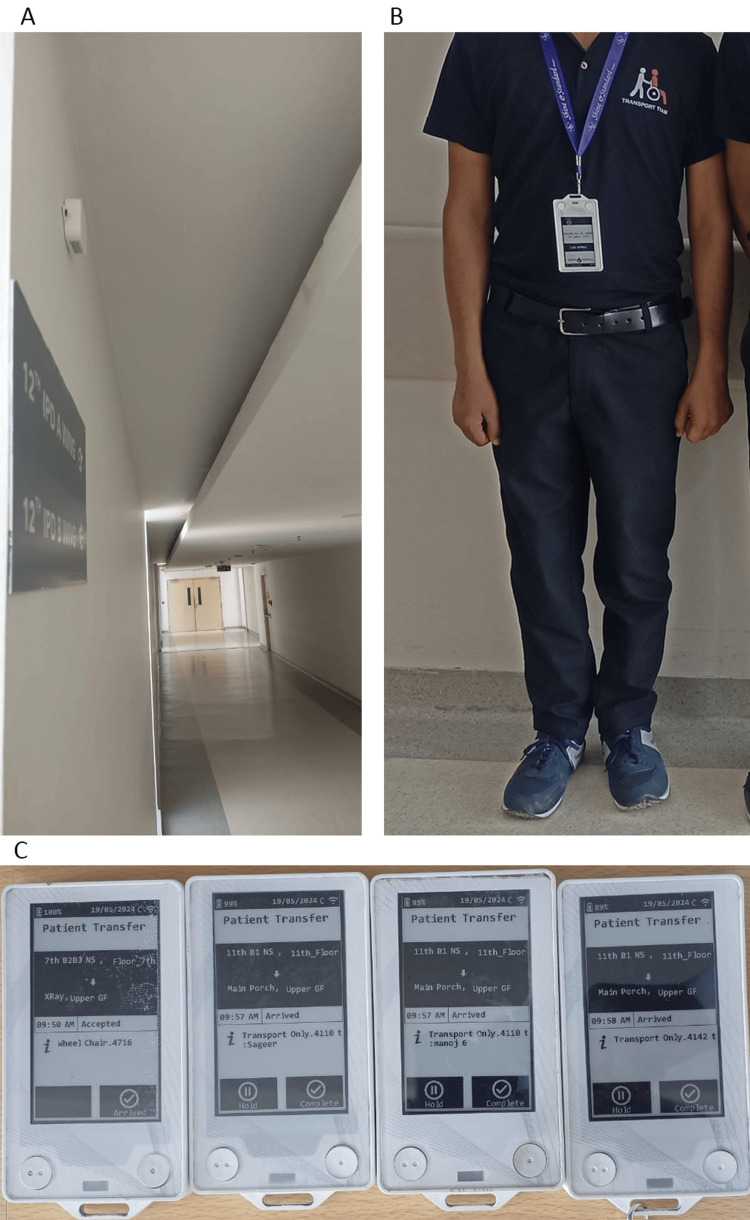
(A) Bluetooth-based beacon mounted on walls. (B) Porter wearing the coaster. (C) Coasters embedded with Bluetooth tags having ID and RSSI. ID: identifier; RSSI: received signal strength indicator.

A Bluetooth beacon-based object tracking system was implemented to automate porter tracking within the hospital. A network of beacons with a 20-meter range was mounted on walls and ceilings to scan and locate porters (Figure 1A). These beacons require minimal maintenance and last up to three years on a single battery. The porters are provided with coasters embedded with a Bluetooth tag having a unique identifier (ID) and received signal strength indicator (RSSI). When porters wearing these coasters move within the range of a beacon, the beacon scans the tag’s ID and RSSI to collect their spatial information (Figures 1B, 1C). These data are transferred back to a remote server to determine the device’s location. All of the coasters are rechargeable and are equipped with long-lasting 2400 mAh batteries and protective silicon covers for longevity.

**Figure 2:**
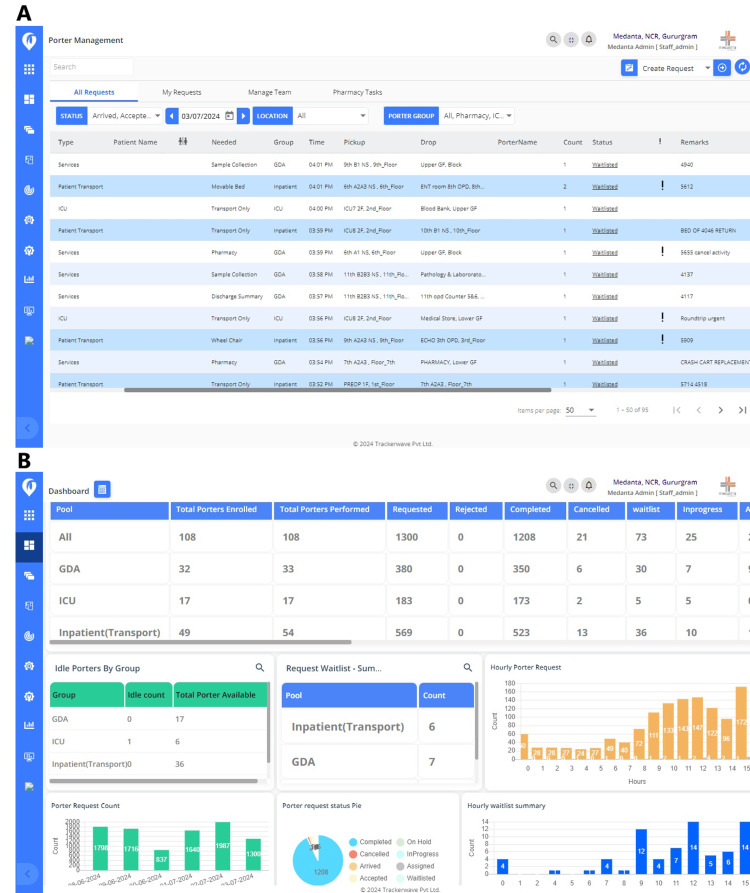
(A) The interface also provides detailed information about the requestor, request description, task type, requirement, request time, request location, destination location, porter name, porter count, and status of the request. (B) Screen with dashboard of IPTS user interface (UI) makes the porter process transparent to the porter supervisor or team leader (TL) by displaying porter request summary by pool (total day), hourly porter request, porter request count, porter request status, hourly waitlist summary, and porter location-wise movement. IPTS: indoor porter tracking system.

The IPTS comprises of two major software components: a dispatch module and a database. When the requester creates a request for the porter service, the following four sets of activities occur: the dispatch module first checks the status of each porter in the database, including their locations and current workloads. The tracking system then provides location information of each porter, based on which the IPTS selects an available porter closest to the requester. This tracking system also tracks the status of each request, as seen in Figure 2A. Alternatively, the porter dispatch process can be manually operated by a manager in the porter center using a screen with the porter dashboard (Figure 2B). After a porter is selected, both the requestor and the porter receive notifications from the IPTS. When the porter completes their assigned task, they inform the IPTS through a very simple one-step user experience in the coaster.

A proof of concept (PoC) was conducted by the deployment of tracking devices in phases, first at the A-wing then the B-wing to monitor the movement of patients to and from the wards to different areas in the hospital, namely, the procedure rooms, ICU, radiology, and the porch/lobby.

The PoC investigated the proficiency of IPTS in reducing the porter workload, streamlining porter management, and the return on investment (ROI) after the technology is implemented.

Capital expenditure and return on investment (ROI)

Capital expenditure for implementation of IPTS within the hospital involves costs for software, training, and maintenance. ROI was evaluated for over three years taking into consideration costs for capital expenditure for implementing the system. Then, the estimated measurable benefits such as reduced labor costs and competitive edge were compared with the ROI to decide the feasibility of system installation and further goals.

## Results

Daily operations of porter services

The field trial conducted for three weeks investigated the workload of porters, peaks in demand, and bottlenecks in efficient porter service management. The hospital receives an estimated total patient movement of 2510 on peak days among which the maximum movement was recorded at the main porch pool (~1335 patients, 53%). The second most prominent patient movement of 969 (39%) was recorded at the inpatient department (IPD), followed by comparatively less patient movement of 206 (8%) during the night.

The three-week investigation reported a total of 1008 patient movements through the main porch of the hospital. Out of 1008, 285 (28%) raised TPT calls for transport from the outpatient department (OPD) or upper ground diagnostic area to the main porch. Out of 285 calls, only 110 (38%) calls were received by the TPTs, while 175 (62%) calls were unanswered due to workload and time management. Among 110 received calls by TPTs, 42 (38%) were allotted to the TPTs from the pool and 68 (24%) were self-received. The assessment of transport tasks from OPD floors to the main lobby revealed only 10% of the total assisted patients were catered to by TPT staff on a daily basis. On peak days, among 860 IPD bed occupancies recorded, only 461 call tickets were serviced. The average wait time to get a porter was five minutes, while the average time required for a porter to complete a request was 15 minutes. The total turnaround time (TAT) for a porter reported at A-wing was 28 minutes and B-wing was 26 minutes.

**Figure 3:**
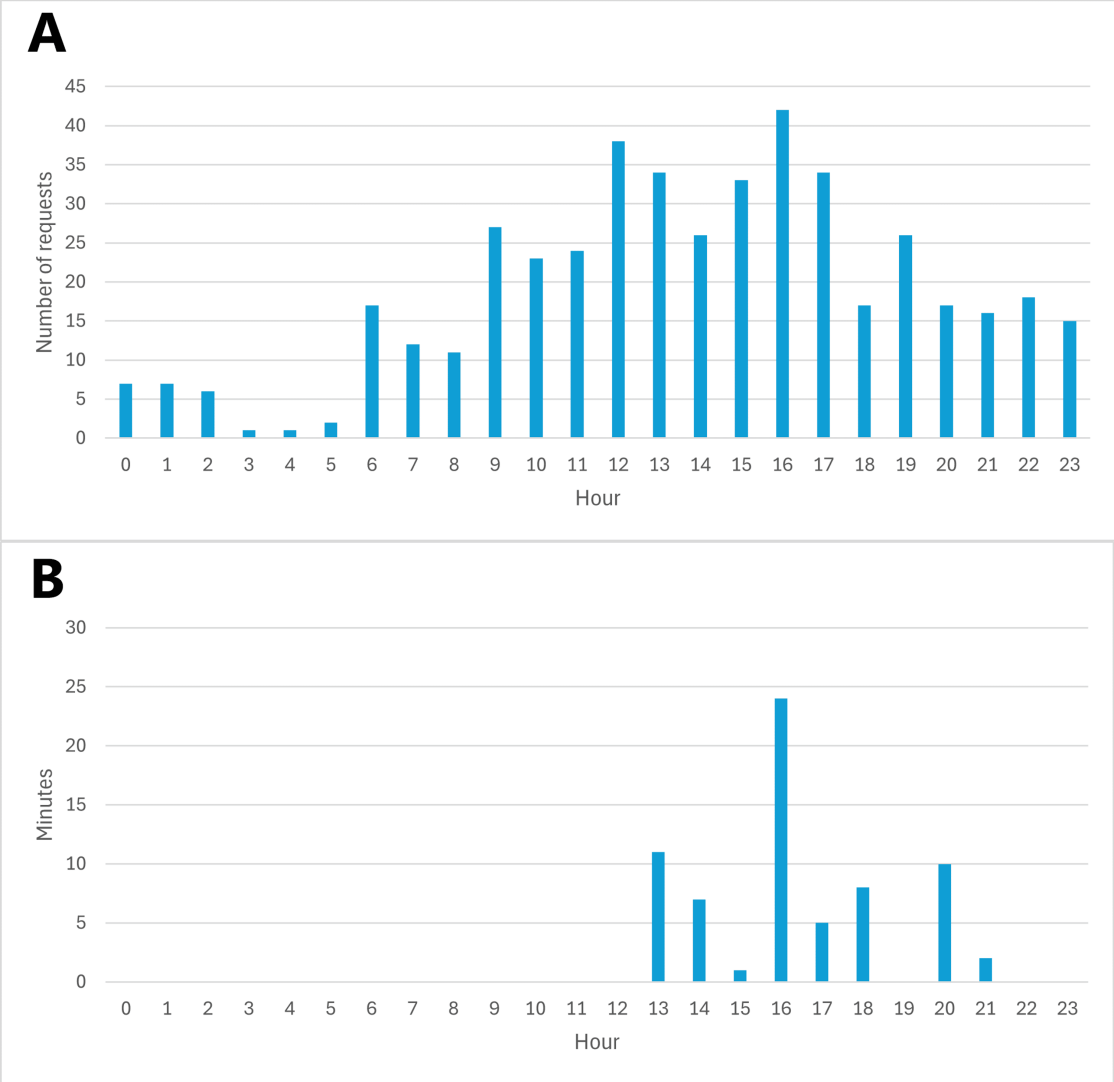
Hourly patient movement monitored over 24 hours in the hospital. (A) The number of porter requests every hour. (B) Time spent by patients waiting for porters.

The hourly patient movement and waiting time over 24 hours revealed high patient movement between 9 am to 7 pm, with a maximum demand peak at 4 pm. Waiting time for patients was observed between 1 pm and 9 pm, with a maximum recorded at 4 pm (Figure 3). The average OPD lift waiting time for wheelchair-assisted patients between floors (e.g., 8th to 11th floor) and from one floor to another via a transition floor (e.g., 3rd to 7th to 11th) was 12 minutes and 19 minutes, respectively.

**Figure 4:**
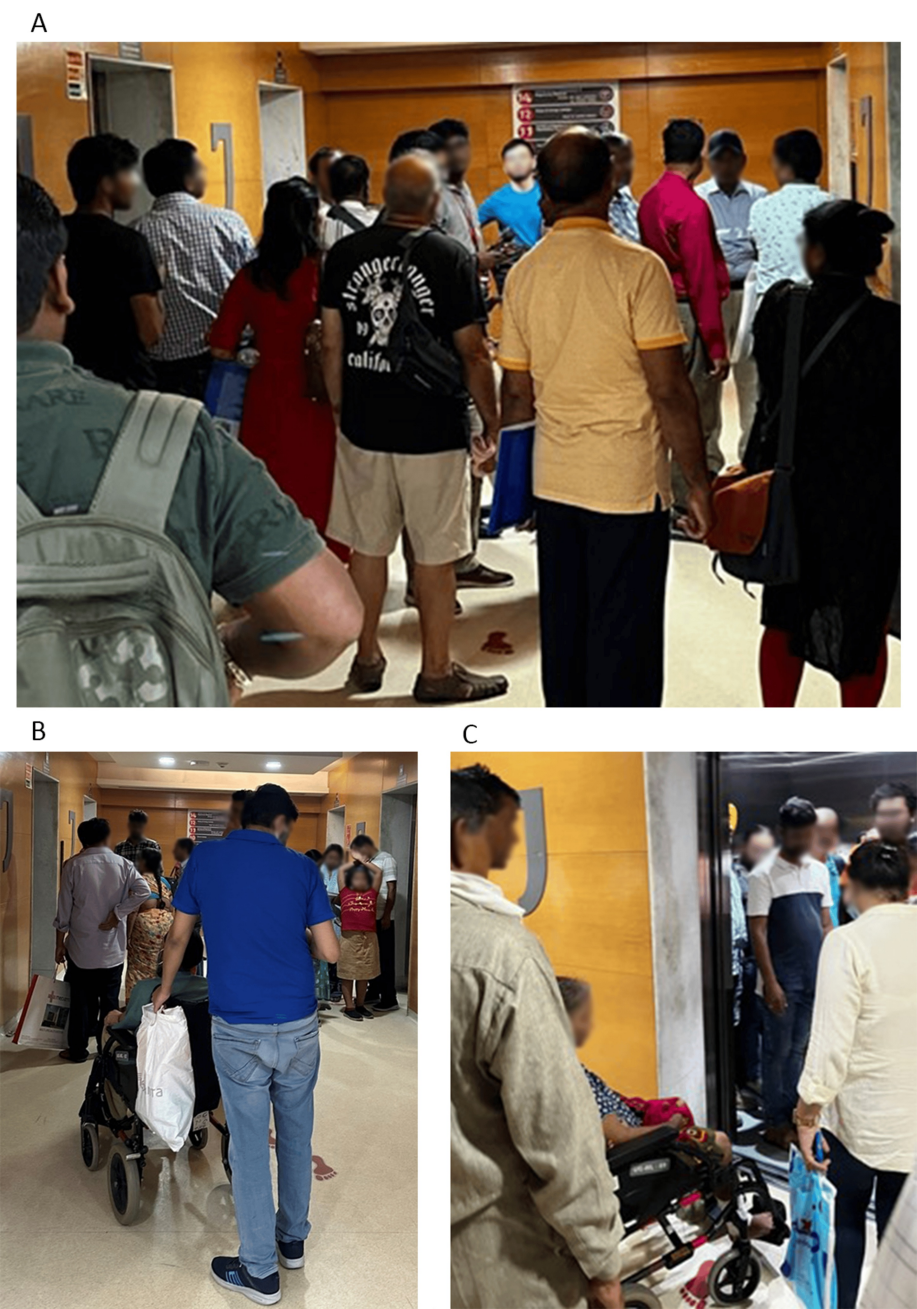
Pain points were identified while transitioning between floors or from service floors to the main lobby. (A) Heavy crowd at peak hours. (B) Unavailability of transport porters for patient care. (C) Overcrowded lifts.

The hotspot areas of long waiting hours and peak crowds were investigated for possible bottlenecks that increase the workload for porters. Pain points were identified at transitions between floors and from various service floors to the main lobby (Figure 4). The pain point investigation highlighted many issues faced by patients such as the unavailability of and high response times for TPTs. Patients and attendants were observed to grow impatient and move wheelchairs via the elevators themselves. Significant crowding was observed during peak hours, with a lack of appropriate crowd management.

Proof-of-concept

The first phase of the PoC was done in the A-wing of the hospital and the implementation was at the near-production level. It enabled the raising of porter requests from any designated stations in the A-wing to IPDs, ICUs, diagnostics, ER, OPD areas, and vice versa.

The base-level staffing of 26 TPTs and two team leaders (TLs) was optimized by two TPTs and one TL. The baseline porter service average TAT of 28 minutes was reduced to ~20 minutes.

After the PoC in the A-wing, it was implemented in the B-wing. The base-level staffing of 31 TPTs and two TLs was optimized by three TPTs and one TL. The baseline porter service average TAT of 26 minutes was reduced to ~18 minutes.

Improvements were noted in productivity measures like number of calls picked, rejected, and timed-out. This also allowed intelligent auto-rerouting or auto-escalation of requests to the next available porters or team leaders in case of unavailability. The requester was able to track the path of the porter improving patient safety. Porters also experienced improved flexibility by being able to self-create tasks to block out time needed for breaks. Data collection was improved for performance assessments of individual porters or larger audits.

Capital expenditure

Healthcare leaders face immense pressure for resource optimization due to disruptive competition, cost containment pressure, and stringent regulatory compliance. An efficient porter system correlates directly with patient care and outcomes; however, effective management is complex due to numerous constantly changing parameters. Improper porter allocation poses various challenges in daily operations and raises concerns about timely patient care. Importantly, a total cost of ownership (TCO) analysis was necessary to determine the cost recovery before the actual deployment of the system.

The deployment of IPTS for managing 100 porters in a hospital setting comes with a three-year TCO of Indian Rupees (INR, ₹) 5.6 million (US$ 67,000). The breakdown of the budget reveals a one-time software cost of ₹2.2 million, annual hardware maintenance of ₹1.0 million, and an annual maintenance contract (AMC) beginning from the 2nd year at ₹200,000 per year. The initial investment will be recovered by optimizing the deployment of nine out of 100 TPTs, leaving 91 TPTs. This optimization anticipates generating monthly savings of ₹180,000 based on an estimated salary of ₹20,000 per TPT per month thus proposing a positive ROI for the hospital by the end of year two (Table 3).

**Table 3:** Detailed budget of capital expenditure for IPTS implementation. AMC: annual maintenance contract; IPTS: indoor porter tracking system; M: million(s).

Item	Cost/savings (in ₹)	Description
Commercial costs for the IPTS software		
One-time software cost	2.2M	Initial purchase of the IPTS software.
Annual hardware maintenance	1.0M	Yearly cost to maintain the IPTS hardware.
Annual maintenance contract (AMC) (year 2 onwards)	0.2M	Annual fee for software maintenance starting from the second year.
Total cost of ownership (TCO) (3 years)	5.6M	Sum of all costs over the three-year period (2.2M + 1.0M per year * 3 years + 0.2M per year * 2 years)
Yearly cost breakdown		
Year 1 cost	3.2M	Software cost + annual hardware and maintenance
Year 2 cost	1.2M	Annual hardware maintenance + annual software AMC
Year 3 cost	1.2M	Annual hardware maintenance + annual software AMC
Savings (optimized porter allocation)		
Average porter cost per month	20,000	Assumed monthly cost of employing a porter.
Monthly savings	0.18M	Cost saved per month due to optimized allocation (20,000/month * 9 porters).
Savings in 2 years	4.32M	Total cost saved in 2 years from optimized allocation (0.18M per month * 24 months).
Savings in 3 years	6.5M	Total cost saved in 3 years from optimized allocation (0.18M per month * 36 months).

Building on the effective and profitable outcomes of the IPTS for porter management, the hospital further envisages deploying IPTS for inpatient (IP) medicine delivery. The current IP medicine delivery team of 18 staff is anticipated to reduce in headcount by three positions. Effective management of IP medicine delivery through IPTS requires the acquisition of 12 additional coasters, including three buffers costing ₹9,600 annually for each device. This expanded IPTS implementation costs ₹6.1 million (US$ 72,000), breaking down into a one-time software cost of ₹2.2 million, an increased annual hardware maintenance cost of ₹1.15 million to support the expanded system, and an AMC for the software of ₹0.2 million starting from the 2nd year. Assuming an average monthly cost of ₹20,000 per porter and an optimization of the deployment of 11 porters out of a base of 110, the IPTS projects to generate monthly savings of ₹ 0.22 million. This translates to a substantial cost reduction of ₹5.3 million (US$ 63,000) in two years and approximately ₹8.0 million (US$ 96,000) over three years (Table 4).

**Table 4:** Detailed budget of capital expenditure to deploy IPTS for IP medicine delivery. IP: inpatient; AMC: annual maintenance contract; IPTS: indoor porter tracking system; M: million(s).

Item	Cost/savings (in ₹)	Description
Additional current headcount (IP medicine delivery)	18	Number of staff currently managing IP medicine delivery.
Expected resource savings (IP medicine delivery)	3	Number of staff positions expected to be saved through IPTS.
Additional cost (IP medicine delivery)	0.12M	Annual cost of additional coasters and buffers for IP medication delivery.
Commercial costs for the IPTS software		
Item	Cost/savings (in ₹)	Description
Commercial costs for the IPTS software		
One-time software cost	2.2M	Initial purchase of the IPTS software for porter management.
Annual hardware maintenance (per year)	1.15M	Yearly cost to maintain the IPTS hardware for porters.
Annual maintenance contract (AMC) (year 2 onwards)	0.2M	Annual fee for software maintenance starting from the second year.
Total cost of ownership (TCO) (3 years)	6.1M	The sum of all costs associated with porter management over the three-year period (2.2M + 1.15M per year * 3 years + 0.2M per year * 2 years)
Yearly cost breakdown		
Year 1 cost	3.35M	Software cost + annual hardware maintenance for porters.
Year 2 cost	1.35M	Annual hardware maintenance for porters + annual software AMC
Year 3 cost	1.35M	Annual hardware maintenance for porters + annual software AMC
Savings (optimized porter allocation)		
Average porter cost per month	20,000	Assumed monthly cost of employing a porter.
Number of porters optimized	11	Expected number of porters whose allocation will be optimized (2 additional on a base of 110).
Monthly savings	0.22M	Cost saved per month due to optimized allocation (20,000 per month * 11 porters).
Savings in 2 years	5.3M	Total cost saved in 2 years from optimized allocation (0.22M per month * 24 months).
Savings in 3 years	8.0M	Total cost saved in 3 years from optimized allocation (0.22M per month * 36 months).

## Discussion

In this work, the traditional porter management was evaluated for possible bottlenecks that affected the timely management of porter services. A need for improvement of the traditional ways was highlighted as 62% of porter requests were not answered due to porter workload. The TTT for porters transporting patients in each wing of the hospital was more than 20 minutes. Pain points were identified at transitions between floors or between service floors and the main lobby. The increasing workload on porters directly caused the unavailability of TPTs, crowded elevators, and crowd management thus impacting the patients' quality of care. Studies across hospitals have shown patients' site of call as the major contributing factor for the transport delay [11].

Implementation of IPTS reduced the TTT to 20 minutes and below. The utility of porter staff was improved, and the porter optimization reduced the base levels of staff. The capital expenditure on IPTS installation and maintenance was recouped with a monthly savings of ₹0.18 million. Further vision on expanding the IPTS in IP medicine delivery anticipated a substantial cost reduction of ₹8.0 million over three years. A simulation exercise on contact tracing using RTLS in a tertiary hospital setting reduced time taken by 96.2%, required manpower by 97.6%, and manpower hours required by 97.5% [12]. Similarly, the implementation of a computerized scheduling tool at Mayo Clinic improved staff efficiency [13].

Beacons and Wi-Fi access points provide a scalable foundation for future advancement. The potential uses of RTLS are not only limited to the porter services but also for patient tracking and locating hospital equipment [14]. Implementation of RTLS on mobile otoscopes or ophthalmoscopes in emergency departments (EDs) reduced the locating time to 25 seconds. The ED providers also reported less frustration and burnout levels as they could locate the equipment in a short time span [15]. By leveraging the existing infrastructure, healthcare facilities can readily implement new functionalities without incurring significant additional costs.

Limitations

The present study, being a PoC, was carried out over a short period of six months. Further studies are required to be conducted in larger sample sizes and across various hospital settings to strengthen the generalizability of the findings. A study to see the long-term effect of IPTS monitoring is required to be performed to evaluate the acceptance and satisfaction among patients and healthcare staff. Also, the IPTS requires assessment for its accuracy of detection and signal quality as the hospital environment may increase signal interference.

## Conclusions

An IPTS unlocks a wide range of operational enhancements as well as a rapid ROI. It ensures timely patient transport for discharges and investigational and surgical procedures thus improving TAT and efficient patient care. Real-time tracking of transport staff minimizes patient wait time, facilitates better peak load management, and promotes certainty in the service model. This efficiency translates to better peak load management. The ROI from reduced wait times and improved staff utilization is a compelling reason to invest in IPTS. Additionally, the cost savings from staff optimization lay the groundwork for future technology integrations and positioning healthcare facilities for long-term operational advancements.
